# Identification of RNA silencing components in soybean and sorghum

**DOI:** 10.1186/1471-2105-15-4

**Published:** 2014-01-04

**Authors:** Xiang Liu, Tao Lu, Yongchao Dou, Bin Yu, Chi Zhang

**Affiliations:** 1 School of Biological Sciences & Center for Plant Science and Innovation, University of Nebraska, Lincoln, NE 68588, USA; 2 Shanghai Chenshan Plant Science Research Center, Chinese Academy of Sciences (CAS), Shanghai Chenshan Botanic Garden, 3888 Chenhua Road, Songjiang, Shanghai 201602, China

## Abstract

**Background:**

RNA silencing is a process triggered by 21–24 small RNAs to repress gene expression. Many organisms including plants use RNA silencing to regulate development and physiology, and to maintain genome stability. Plants possess two classes of small RNAs: microRNAs (miRNAs) and small interfering RNAs (siRNAs). The frameworks of miRNA and siRNA pathways have been established in the model plant, *Arabidopsis thaliana* (Arabidopsis).

**Results:**

Here we report the identification of putative genes that are required for the generation and function of miRNAs and siRNAs in soybean and sorghum, based on knowledge obtained from Arabidopsis. The gene families, including DCL, HEN1, SE, HYL1, HST, RDR, NRPD1, NRPD2/NRPE2, NRPE1, and AGO, were analyzed for gene structures, phylogenetic relationships, and protein motifs. The gene expression was validated using RNA-seq, expressed sequence tags (EST), and reverse transcription PCR (RT-PCR).

**Conclusions:**

The identification of these components could provide not only insight into RNA silencing mechanism in soybean and sorghum but also basis for further investigation. All data are available at http://sysbio.unl.edu/.

## Background

Small RNAs, in particular, 20- to 24-nucleotide (nt) in length, belong to two classes: microRNAs (miRNAs) and short interfering RNAs (siRNAs). MiRNAs are regulators of gene expression and affect many biological processes, such as development and physiology in plants and animals [1-3]. Their dysregulation often causes developmental defects and diseases of plants and animals. MiRNAs are released as a duplex from an imperfect step-loop, which resides in the miRNA primary transcripts (pri-miRNA) [1-3]. SiRNAs are chemically indistinguishable with miRNAs but they originate from long perfect double-stranded RNAs (dsRNAs) [2,3]. Plants encode several classes of siRNAs including siRNAs derived from repetitive DNAs (ra-siRNAs) and transacting siRNAs (ta-siRNAs) [2,3]. Ra-siRNAs regulate gene expression at transcriptional levels by directing DNA methylation at homologs loci through a process named RNA-directed DNA methylation (RdDM) [2,3]. In contrast, ta-siRNAs act like miRNAs to regulate gene expression at post-transcriptional levels [3]. The framework of plant miRNA/siRNA biogenesis and function has been established in *Arabidopsis thaliana* (Arabidopsis); several different categories of genes are involved in the pathways for their generations and loading.

In Arabidopsis, the generation of miRNAs and siRNAs requires the DICER-LIKE proteins (DCL) [2]. DCLs are the RNAase III enzymes that cut the dsRNAs to release ~ 22 nt RNA duplexes, which have 2 nt 3′ overhangs at each end [1]. Arabidopsis encodes four DCLs: DCL1, DCL2, DCL3, and DCL4. DCL1, which associates with HYL1, a dsRNA binding protein, and SERRATE (SE), a zinc protein, cuts pri-miRNAs two times to release 21 nt miRNA duplex in nucleus [4-6]. DCL2 is responsible for 22 nt viral-derived siRNAs when plants are infected [7]. DCL3 generates 24 nt ra-siRNAs and DCL4 produces 21 nt ta-siRNAs and some miRNAs [8-10]. The generation of both miRNAs and siRNAs also requires the single-stranded RNA (ssRNA)-binding proteins DAWDLE and TOUGH [11,12]. After generation, miRNA and siRNA duplexes are 2′–O-methylated at 3′-terminal nucleotide by a dsRNA methylase HEN1 [13]. The methylation protects miRNAs from degradation and 3′ untemplated uridine addition [14]. The Arabidopsis HASTY (HST) gene is an ortholog of the human exportin 5 gene. After generation, miRNAs are exported to cytoplasm by HST-dependent or independent pathways [15], where they function. Interestingly, some components of miRNA biogenesis pathway are also targets of miRNAs. For example, in soybean, miR1515 can target DCL2 and leads to hypernodulation [16,17].

RNA-dependent RNA polymerase (RDR) is another essential player for siRNA production. Among six RDRs in Arabidopsis, RDR2 converts ssRNAs generated from repetitive DNAs to precursor dsRNAs of ra-siRNAs [8], while RDR6 produces the ta-siRNA precursors [18]. The generation of ra-siRNA also requires a plant specific DNA-dependent RNA polymerase IV (Pol IV) [19-21]. Pol IV is a Pol II-derived plant specific polymerase. It contains many identical subunits of Pol II [22], but the largest subunit NRPD1 and the second largest subunit NRPD2/NRPE2 of pol IV are paralogous of their counterparts in Pol II [22]. Over 90% siRNAs require Pol IV for their production [23]. Pol IV is thought to transcribe ssRNAs that serve as templates of RDR2 from RdDM target loci [19-21]. Another plant specific DNA dependent RNA polymerase V (Pol V) also plays crucial roles in the RdDM pathway [21,24]. Pol V shares eight subunits with Pol IV including NRPD2/NRPE2 [22,25], while NRPE1 (the largest subunit) and other three subunits are distinct from their counterparts in Pol IV [22,25]. Pol V associates with RdDM target loci and produces ~200 nt non-coding transcripts from surrounding regions of some RdDM loci.

MiRNAs and siRNAs are loaded onto the ARGONAUTE (AGO) proteins, which performs target mRNA cleavage and/or translational inhibition, or directs chromatin modification such as DNA methylation [1]. By recognizing the complementary sequences in the targets, miRNA,s and siRNAs guide AGO to silence specific genes [1]. In general, there are multiple AGO genes in a plant species. Arabidopsis possesses 10 AGOs [26], based on sequence similarities, which are grouped into three clades: AGO1, AGO5 and AGO10 belong to the first clades; AGO2, AGO3 and AGO7 compose the second clades; and AGO4, AGO6, AGO8 and AGO9 are within the third clades [26]. AGO1 associates with miRNAs and some siRNAs such as ta-siRNAs to cleave target mRNA and/or inhibit translation [27]. AGO10 specifically sequesters miR166/165 from AGO1, which is essential for shoot apical meristem development [28,29]. AGO7 binds miR390 to cleave the precursor RNA of ta-siRNAs [30]. AGO4, AGO6, and AGO9 majorly bind 24 nt ra-siRNAs to direct DNA methylation [31], but seem to have different target preference [31-33]. It has been proposed that Pol V may recruit AGO4-siRNA complex to RdDM targets though its physical interaction with AGO4 and/or the interaction between its nascent transcripts and AGO4/6 associated siRNAs [34-36]. Recently, 19 and 18 AGOs were identified in rice and maize, respectively [37,38].

In soybean and sorghum, our knowledge on RNA silencing mechanism is still poor. Taking advantage of available genome information and the conservation of RNA silencing components in different plant species, in this study, putative RNA silencing components, including DCL, HEN1, SE, HYL1, HST, RDR, NRPD1, NRPD2/NRPE2, NRPE1, and AGO, are identified in soybean and sorghum. The identification of these components could provide insight into RNA silencing mechanism in soybean and sorghum as well as basis for further investigation.

## Results

### DCLs

**Figure 1:**
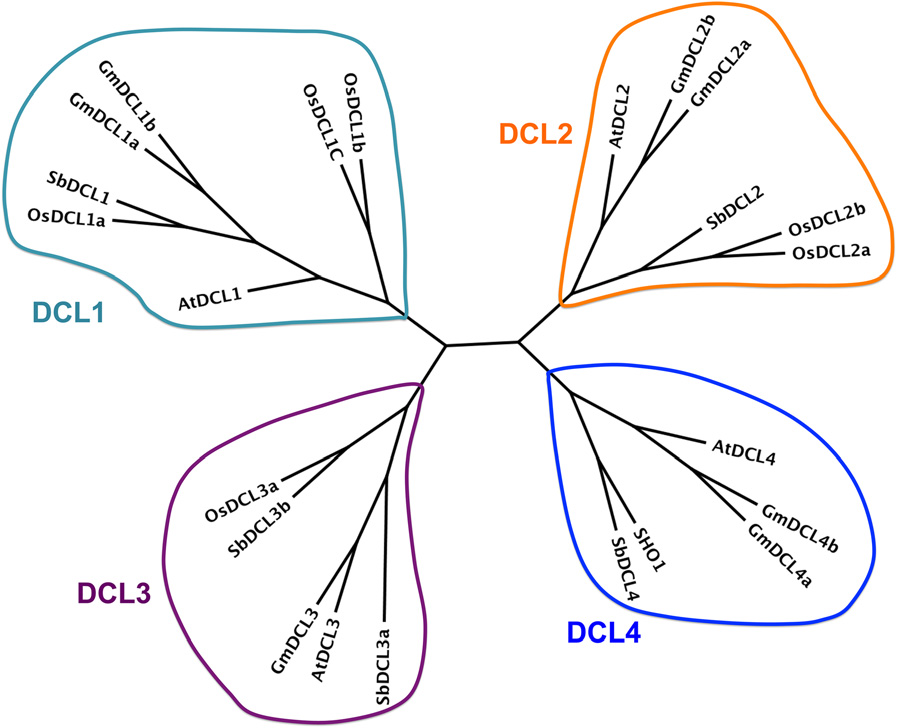
**Phylogenetic tree of DCL genes in soybean, sorghum, Arabidopsis, and rice.** There are two DCL1 in soybean, sorghum, and rice, while only one in Arabidopsis. There are two DCL2 in soybean and rice, but one in sorghum and Arabidopsis. Soybean, Arabidopsis, and rice have only one DCL3, whereas Sorghum has two. Only soybean has two DCL4 and the other has only one DCL4.

The domains of DExD-helicase, helicase-C, Duf283, PAZ, RNase III, and double-stranded RNA-binding (dsRB) are conserved in plant and animal DCLs [39]. Therefore, DCL genes in soybean and sorghum can be identified by searching genes whose proteins have these domains combined with a structure like DCLs in Arabidopsis. The protein domain identification was accomplished with Hidden Markov Models (HMMs). Using HMM analysis to the whole genomes combined sequence similarity search with TBLASTN, 7 and 3 DCLs were identified in soybean and sorghum, respectively (Table 1). Phylogenetic analysis assigns two DCL1, two DCL2, one DCL3, two DCL4 in soybean and one DCL2, two DCL3 in sorghum (Figure 1). These genes are named by using prefix Gm (*Glycine max*) or Sb (*Sorghum Bicolor*) to reflect the species in which they are present and the numbers of their Arabidopsis orthologs, for example GmDCL1 for soybean DCL1 gene. In this manuscript, prefix At (*Arabidopsis thaliana*) and Os (*Oryza sativa*) are used for Arabidopsis and rice, respectively. If there are more than one orthologs, a letter is attached according to the sequence similarity. For instance, the one having the highest similarity with their Arabidopsis ortholog is designed as “a”. If two proteins are identical, they are named as “a” or “b” based on the order of chromosome location numbers: the gene is “a” if it is on the chromosome with a smaller number and the other is “b”. The same nomenclature is used for other RNA silencing components described in the following sections. The HMM search failed to find DCL1 and DCL4 in sorghum annotated genes, and hence, TBLASTN was performed to search AtDCL1 and AtDCL4 protein sequences against the sorghum genome sequence. This approach identified the SbDCL1 from an unannotated region in the chromosome one. This locus is named as Sb01g049105 because it is located between loci Sb01g049100 and Sb01g049110. The expression of SbDCL1 was confirmed by RT-PCR. No DCL4 homolog was identified by TBLASTN, but three predicted proteins, Sb06g022180, Sb06g022190, and Sb06g022200, show similarities to different portions of AtDCL4 from C-terminus to N-terminus. This indicates that these three predicted proteins might belong to one transcription unit. In fact, annotation to the region of these three genes predicts one transcript that encodes a 1630 amino acid (AA) long protein. This new predicted protein, named as SbDCL4, has 63% and 83% similarities to AtDCL4 and OsDCL4, respectively. The soybean DCL1, DCL2, and DCL4 have been duplicated once in the genome and there are high similarities between duplicates. In contrast, only DCL3 has duplicates in the sorghum genome. The GmDCLs are more similar to AtDCLs than SbDCLs do (Figure 1), presumably due to that both soybean and Arabidopsis are dicot while sorghum is monocot. In fact, SbDCLs are more similar to OsDCLs than to AtDCLs (Figure 1).

**Table 1:** DCL genes in soybean and sorghum

**Soybean**	**Locus names**	**Location coordinates**	**Protein length**^**(a)**^
GmDCL1a	Glyma19g45060	50301996..50314756	1945
GmDCL1b	Glyma03g42290	47521814..47533928	1947
GmDCL2a	Glyma09g02930	2040845..2056497	1414
GmDCL2b	Glyma09g02920	2023553..2036875	1421
GmDCL3	Glyma04g06060	4597237..4617503	1674
GmDCL4a	Glyma13g22450	25949252..25984083	1637
GmDCL4b	Glyma17g11235	8432127..8454206	1636
**Sorghum**	**Locus names**	**Location coordinates**	**Protein length**^**(a)**^
SbDCL1	Sb01g049105	72107638..72118571	1947
SbDCL2	Sb01g015670	15275142..15291284	1385
SbDCL3b	Sb01g018890	19808921..19819749	1586
SbDCL3a	Sb03g043355	70703867..70712978	1651
SbDCL4	Sb06g022180	51402432..51427702	1630

^(a)^Protein lengths were obtained from Phytozome annotations, except SbDCL1 and SbDCL4, whose protein lengths were predicted.

**Figure 2:**
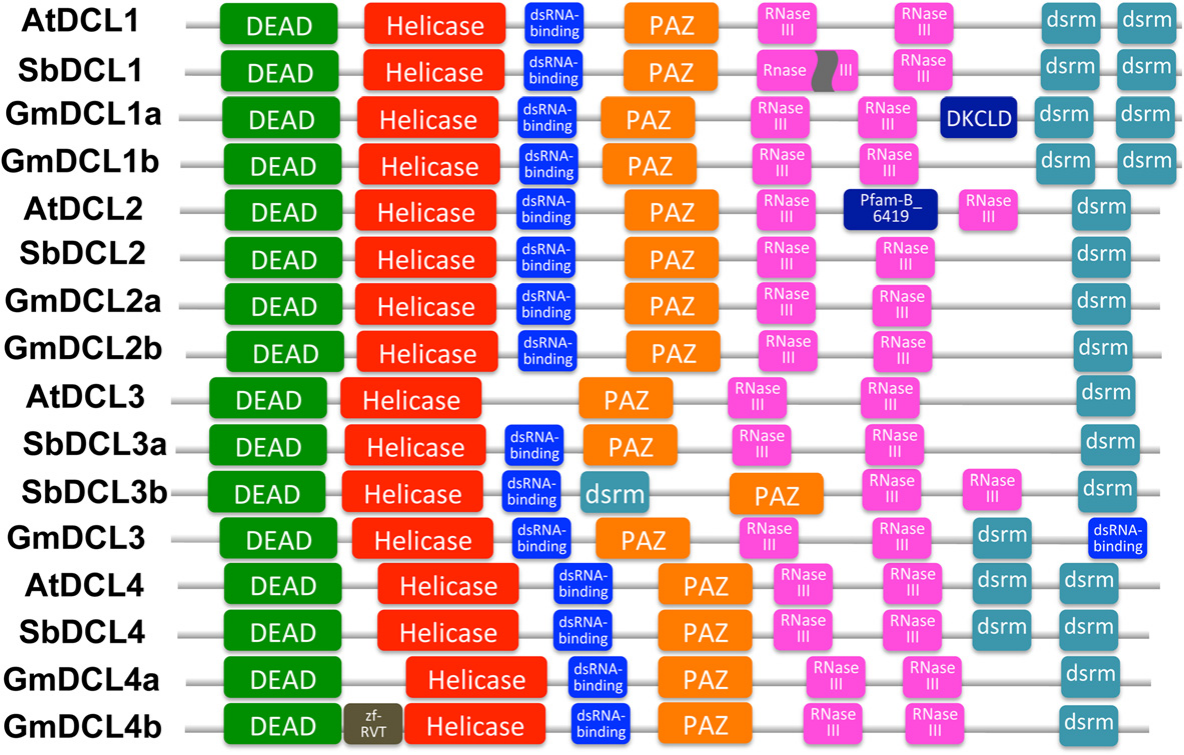
**Domain compositions of DCL genes in soybean, sorghum, and Arabidopsis.** All DCLs have major domains: DExD-helicase, helicase-C, Duf283 (now called dsRNA-binding domain), PAZ, RNase III (two), and dsRB (shown as dsrm in the figure), except AtDCL3, which missed the dsRNA-binding domain. The first RNase III domain in SbDCL1 is separated into two segments, and the first dsrm domain in SbDCL3b appears between dsRNA-binding and PAZ domains.

A Dicer gene and its homologs always contain two RNaseIII domains, termed “a” and “b”, each of which cleaves one strand of a dsRNA. The PAZ domain binds to the 3′-end of a dsRNA [39]. The distance between the PAZ domain and the cleavage site of RNase III domain determines the length of small RNAs [39]. The domain Duf283 is now known to be a dsRNA-binding domain [40]. The protein domains present in soybean and sorghum DCLs are similar to their counterparts in Arabidopsis except some differences in DCL1 and DCL3. The RNase IIIa domain of SbDCL1 is divided into two segments, whereas it is present as an undivided domain in AtDCL1. Duf283 exists in both soybean and sorghum DCL3 while it is absent in AtDCL3. Figure 2 shows the combinations of DCL domains in soybean, sorghum, and Arabidopsis.

### HEN1, SE, HYL1, and HST

By searching AtHEN1 protein sequence against soybean and sorghum genomes with TBLASTN, two soybean and one sorghum HEN1 homologs were identified. Soybean HEN1 from chromosome 5 and 8 are named as GmHEN1a and GmHEN1b, respectively (Table 2). The protein sequences of GmHEN1a/b and SbHEN1 have only 40-50% similarity with AtHEN1. The identical protein sequences of GmHEN1a and 1b suggest a recent duplication event. Like AtHEN1, GmHEN1a/b, and SbHEN1 contained two dsRNA binding domains, a La-motif-containing domain (LCD), a PPIase-like domain (PLD), and a highly conserved methyltransferase (MTase) [41], which indicates that HEN1s act on the miRNA or siRNA duplexes.

**Table 2:** HEN, SE, HYL, and HST genes in soybean and sorghum

**Soybean**	**Locus names**	**Location coordinates**	**Protein length**^**(a)**^
GmHEN1a	Glyma08g08650	6154425..6162878	945
GmHEN1b	Glyma05g25670	31711795..31720368	945
GmSEa	Glyma04g15990	16903215..16912286	711
GmSEb	Glyma06g47265	49778255..49785958	714
GmSEc	Glyma08g24370	18572474..18580257	717
GmHSTa	Glyma19g42930	48730756..48747694	1206
GmHSTb	Glyma03g40320	46101672..46115353	1206
GmHYL1a	Glyma04g10230	8449238..8453316	359
GmHYL1b	Glyma06g10200	7694944..7698509	363
**Sorghum**	**Locus names**	**Location coordinates**	**Protein length**^**(a)**^
SbHEN1	Sb02g003680	4115195..4120227	942
SbSEa	Sb10g028740	58608434..58614520	713
SbSEb	Sb07g026820	61961292..61971961	642
SbSEc	Sb04g003540	3375268..3380019	796
SbHSTa	Sb03g014460	19484723..19502625	1201
SbHSTb	Sb05g018936	45814283..45835954	1267
SbHYL1	Sb08g000900	828502..830795	394

^(a)^Protein lengths were obtained from Phytozome annotations.

Soybean and sorghum genomes each encode three Arabidopsis SE homologs (Table 2). AtSEs have around 75% and 50-67% sequence similarities to GmSEs and SbSEs, respectively. Same as AtSEs, soybean and sorghum SEs possess an N-terminal unstructured region followed by an N-terminal domain containing several nuclear localization signals, a middle-domain, a core Zinc-finger domain, and a C-terminal unstructured region [42]. Although similarities among GmSEs and among SbSEs are around 90%, their N-terminal unstructured regions (1–92 AA) are not conserved, which is consistent with the fact that the N-terminal unstructured region of a SE is not essential for its function in miRNA metabolism [42].

Both soybean and sorghum genomes encode two Arabidopsis HST homologs (Table 2). Although GmHSTa and b proteins are 79% similar to AtHST, they are 96% similar to each other. SbHSTa protein is 73% similar to AtHST, but SbHSTb shows only 59% similar to SbHSTa and 49% to AtHST. The low similarities of SbHSTb with SbHSTa and AtHST indicate that SbHSTb might be evolved into novel functions besides exporting miRNAs. Further research is deserved to conduct to test this hypothesis.

The dsRNA binding protein HYL1, which contains two dsRNA-binding domains at its N-terminus, is another essential component of miRNA biogenesis [43]. Soybean genome encodes two HYL1 homologs that are 96% similar to each other, whereas sorghum encodes one HYL1 homolog (Table 2). GmHYL1a/b and SbHYL1 have more than 70% sequence identity with AtHYL1 at their N-terminal regions (~220 AA), which contains two dsRNA-binding domains. However, their C-terminal regions have no or little homology to that of AtHYL1. This is consistent with the fact that two dsRNA domains of HYL1 are essential and sufficient for its activity in miRNA biogenesis [44].

### RDRs

**Figure 3:**
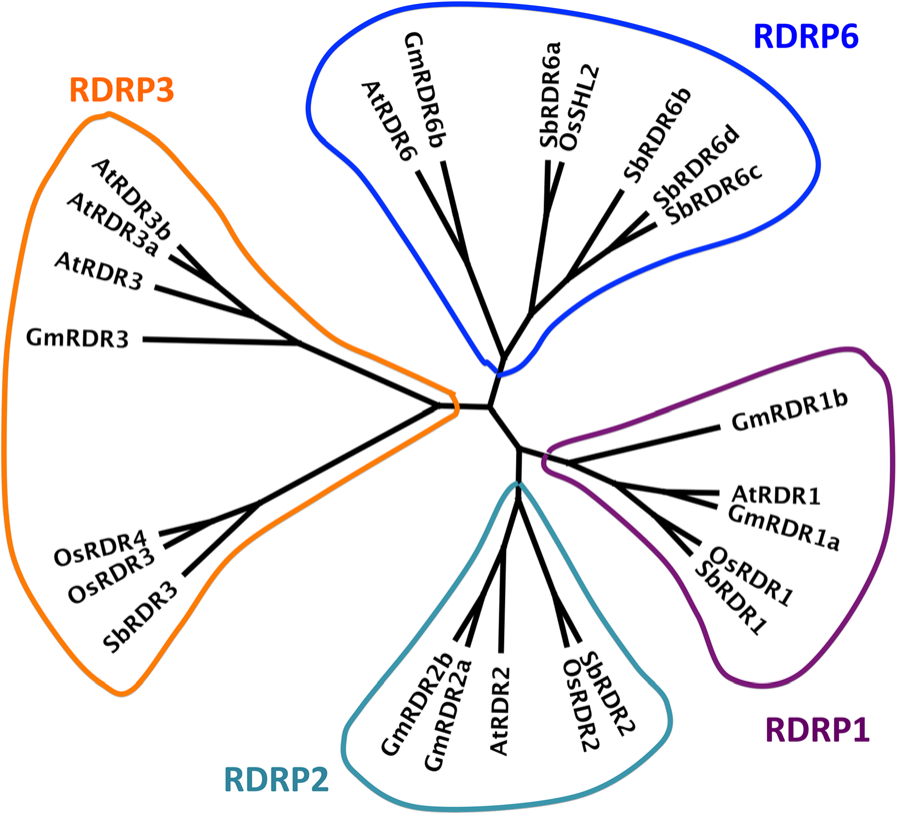
**Phylogenetic tree of RDR genes in soybean, sorghum, Arabidopsis, and rice.** All have one RDR1 and RDR2 genes, except soybean, which has two of each. Arabidopsis has three RDR3s, rice has two RDR3s, whereas soybean and sorghum have only one RDR3. While soybean, Arabidopsis, and rice have only one RDR6, sorghum has four RDR6s.

RDR is another important component of gene silencing, and it has a conserved RDRP domain. Six RDRs in Arabidopsis can be divided into four families: RDR1, RDR2, RDR3, and RDR6 [45,46]. RDR3 family contains three members (RDR3a-c; also known as RDR3, 4 and 5), which share more than 80% similarities to each other [45]. All proteins in soybean and sorghum were scanned for the RDRP domain with HMM, and the candidates were compared the results from searching all Arabidopsis RDR protein sequences against soybean and sorghum genomes with TBLASTN. Soybean and sorghum each encodes seven RDRs, which can be grouped into four families as Arabidopsis (Table 3). In soybean, RDR1, 2, and 6 families each contains two members and RDR3 family has a single gene, which is more similar to RDR3b in Arabidopsis. In sorghum, RDR1, 2 and 3 families each contains one member and the RDR6 family possesses four. The Phylogenetic tree of these RDR genes is shown in Figure 3.

**Table 3:** Pol IV and Pol V genes in soybean and sorghum

**Soybean**	**Locus names**	**Location coordinates**	**Protein length**^**(a)**^
GmRDR1a	Glyma02g09470	7412417..7422912	1125
GmRDR1b	Glyma07g26900	29724547 - 29732281	1135
GmRDR2a	Glyma05g02000	1446199..1451446	1121
GmRDR2b	Glyma17g09920	7392558..7398047	1120
GmRDR3	Glyma01g01210	841604..854009	988
GmRDR6a	Glyma04g07151	5561044..5567083	1204
GmRDR6b	Glyma06g07251	5276132..5282404	1204
**Sorghum**	**Locus names**	**Location coordinates**	**Protein length**^**(a)**^
SbRDR1	Sb04g028630	58770228..58774820	1114
SbRDR2	Sb06g019330	48882239..48887912	1128
SbRDR3	Sb03g002800	2693800..2705322	1127
SbRDR6a	Sb03g022880	45335348..45342015	1207
SbRDR6b	Sb07g006150	8813848..8818345	1220
SbRDR6c	Sb10g025950	55293576..55298021	1239
SbRDR6d	Sb10g025970	55301370..55306226	1251

^(a)^Protein lengths were obtained from Phytozome annotations.

**Figure 4:**
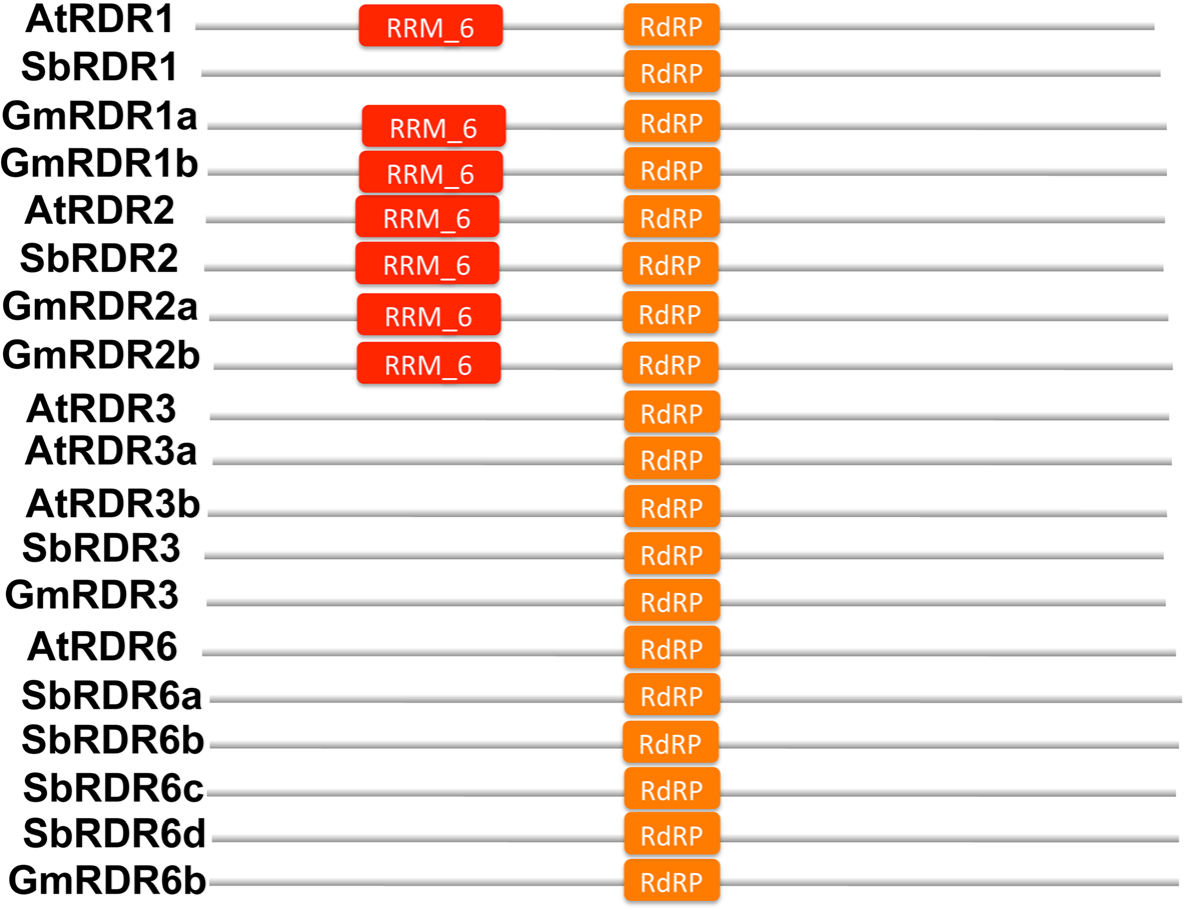
**Domain compositions of RDR genes in soybean, sorghum, and Arabidopsis.** All RDR genes have an RDRP domain, while RDR1s and RDR2s have an additional RNA recognition motif (RRM_6).

Like other RDRs, these sorghum and soybean RDRs contain a common sequence motif corresponding to the catalytic β’ subunit of DNA-dependent RNA polymerases [46]. The putative catalytic domains of soybean and sorghum RDR1, 2, and 6 proteins all contain the DLDGD motif, which is highly conserved in other identified RDRs [46]. Like RDRs in other plants [46], RDR1, 2, and 6, proteins in soybean and sorghum also have the conserved subsequences, CSGS, GSGG, and ASGS, before the DLDGD motif. Protein sequence analysis shows that the second position on the DLDGD motif has some variations. Like AtRDR3, the motif sequences in soybean and sorghum RDR3 proteins are DFDGD [46]. There are two more conserved motifs in all RDR proteins. All RDR1, 2, and 6 sequences including soybean and sorghum, carry a PCLH(P/S)GD(V/I)R motif while RDR3 has PGLH(F/P)GDIH [46]. The second motif is A(V/L/I)DxPKxG; proteins for RDR1, 2 and 6 genes specifically have AVD(F/S)(P/A)KTG motif and RDR3 proteins have A(L/I)DAPKxG [46]. Like other plants, RDR1, 2, and 6 proteins in soybean and sorghum also have two additional conserved motifs: (A/T)(F/Y)QIRY and ASAWY [46]. Figure 4 shows the combination of domains in RDRs in soybean, sorghum, and Arabidopsis.

### Soybean and sorghum Pol IV and Pol V

In order to gain insight into the Pol IV and Pol V complex, the largest and the second largest subunits of Pol IV are searched by searching AtNRPD1, AtNRPE1, and AtNRPD2/NRPE2 against soybean and sorghum genomes with TBLASTN. Soybean encodes two NRPD1, two NRPD2/NRPE2, and two NRPE1, and hence they are named as GmNRPD1a/b, GmNRPD2a/NRPE2a, GmNRPD2b/NRPE2b, and GmNRPE1a/b. Sorghum encodes one NRPD1, one NRPD2/NRPE2, and one NRPE1 for SbNRPD1, SbNRPD2/NRPE2, and SbNRPE1. All genes are listed in Table 4. GmNRPD1a is 97% similar to GmNRPD1b and both are around 67% similar to AtNRPD1, whereas GmNRPD2a/NRPE2a and GmNRPD2b/NRPE2b are 98% similar to each other and are 80% similar to AtNRPD2/NRPE2. GmNRPE1a and GmNRPE1b share 79% similarity and are 61% similar to AtNRPE1. SbNRPD1, SbNRPD2/NRPE2, and SbNRPE1 show only 51% homolog with their Arabidopsis counterparts. Phylogenetic analysis shows the close evolutionary relationships of NRPD1/NRPE1 to RPB1 and NRPD2/NRPE2 to RPB2 in both soybean and sorghum, which agrees with the proposed mode that NRPD1/NRPE1 and NRPD2/NRPE2 are alleles to RPB1 and RPB2, respectively [21]. Protein sequence alignment also revealed the presence of conserved catalytic center residues within NRPD1s and NRPE1s in soybean and sorghum.

**Table 4:** NRPD genes in soybean and sorghum

**Soybean**	**Locus names**	**Location coordinates**	**Protein length**^**(a)**^
GmNRPD1a	Glyma11g02921	1899781..1914267	1454
GmNRPD1b	Glyma01g42480	53715626..53729704	1457
GmNRPE1a	Glyma15g37710	43587734..43604368	2098
GmNRPE1b	Glyma13g26691	29874222..29890995	2020
GmNRPD2a/GmNRPE2a	Glyma06g06480	4630158..4635863	1205
GmNRPD2b/GmNRPE2b	Glyma04g06440	4917151..4922485	1205
**Sorghum**	**Locus names**	**Location coordinates**	**Protein length**^**(a)**^
SbNRPD1	Sb06g025933	54997978..55015850	1203
SbNRPE1a	Sb03g046922	73928743..73935629	1571
SbNRPD2a/SbNRPE2a	Sb01g042100	65423793..65438741	1228
SbNRPD2b/SbNRPE2b	Sb06g030300	58746699..58757600	1239

^(a)^Protein lengths were obtained from Phytozome annotations.

### AGO proteins

**Figure 5:**
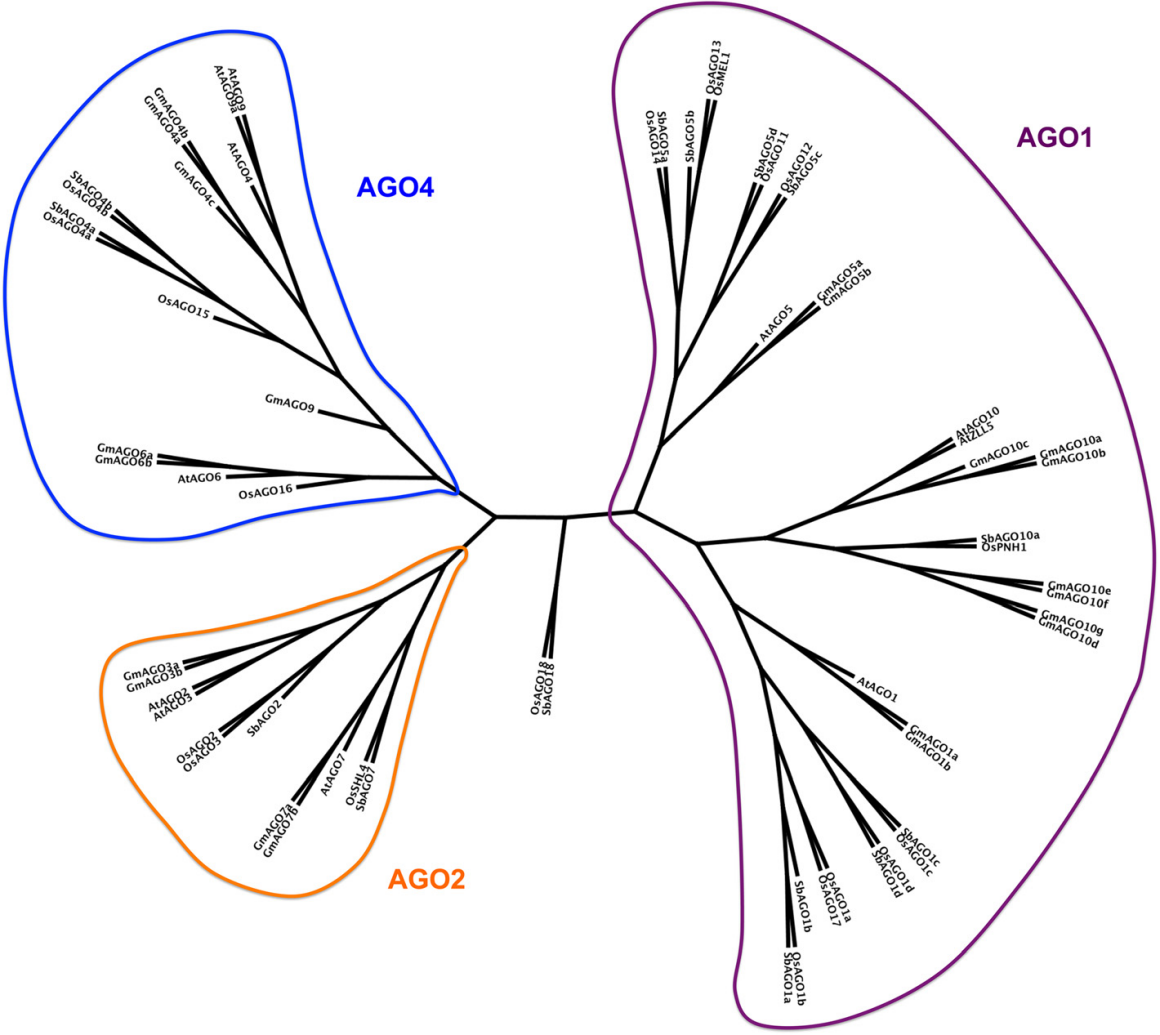
**Phylogenetic tree of AGO genes in soybean, sorghum, Arabidopsis, and rice.** There are 35, 12, and 16 genes in AGO1, AGO2, and AGO4 families, respectively. Sorghum and rice have one AGO18, respectively.

AGO proteins often contain four domains: N-terminal function-unknown domain (pfam DUF1785), PAZ, MID, and C-terminal PIWI domains. Proteins in soybean and sorghum with these four domains are identified by HMM analysis, and TBLASTN was performed to align Arabidopsis AGO proteins against sorghum and soybean genomes for comparison. Forteen AGOs in sorghum and 21 AGOs in soybean were identified, respectively (Table 5). Based on phylogenetic analysis, all AGO proteins can be grouped into three families: AGO1, AGO2, and AGO4. For sorghum, the AGO1 family consists of 10 members, who are four AGO1s, four AGO5s, one AGO10s and one AGO18, which is named with OsAGO18 because of their high similarity [38]. The AGO2 family has two proteins, AGO2 and AGO7, and the AGO4 family contains two AGO4 proteins. In soybean, 11 soybean AGOs are grouped as AGO1 family: two clustered to form the AGO1 subfamily, two for the AGO5 subfamily, and seven for the AGO10 subfamily. Among four soybean AGO proteins in the AGO2 family, two are clustered with AGO2/3 and the others are more closely related to AGO7. Two genes in AGO2/3 subfamily are named as GmAGO3 because they are more similar to AtAGO3 than AtAGO2. The soybean AGO4 family has six members: three AGO4s, two AGO6s, and one AGO9. Like DCLs, these AGO proteins are named based on their similarities with their Arabidopsis counterparts (Figure 5). In the current genome annotation, GmAGO10g and GmAGO10e were predicted to encode 671 and 729 AA-long proteins, respectively, which miss C-terminal portions of PIWI domains. Additional gene annotation procedure was conducted and finds that AGO10g and AGO10e may encode two longer proteins with 909 AA and 908 AA, respectively.

**Table 5:** AGO genes in soybean and sorghum

**Soybean**	**Locus names**	**Location coordinates**	**Protein length**^**(a)**^
GmAGO1a	Glyma16g34300	36927742..36935147	1053
GmAGO1b	Glyma09g29720	36540972..36548174	1058
GmAGO3a	Glyma20g02820	2427591..2432094	966
GmAGO3b	Glyma15g13260	9894903..9900077	1037
GmAGO4a	Glyma14g04510	3084871..3092810	906
GmAGO4b	Glyma02g44260	48876494..48884832	906
GmAGO4c	Glyma20g12070	16970965..16979785	947
GmAGO5a	Glyma12g08860	6617948..6624348	961
GmAGO5b	Glyma11g19650	16394576..16400897	890
GmAGO6a	Glyma13g26240	29451174..29461142	913
GmAGO6b	Glyma15g37170	42791338..42795410	779
GmAGO7a	Glyma01g06370	6606760..6612290	1029
GmAGO7b	Glyma02g12430	10698404..10703841	1031
GmAGO9	Glyma06g47230	49737236..49744788	881
GmAGO10a	Glyma20g28970	37910755..37919552	974
GmAGO10b	Glyma10g38770	46551836..46561789	974
GmAGO10c	Glyma02g00510	290702..300214	972
GmAGO10d	Glyma06g23920	21314534..21331539	909
GmAGO10e	Glyma05g08170	8130422..8137850	908
GmAGO10f	Glyma17g12850	9707144..9714751	904
GmAGO10g	Glyma04g21450	24593027..24608490	909
**Sorghum**	**Locus names**	**Location coordinates**	**Protein length**^**(a)**^
SbAGO1a	Sb06g025560	54517079..54525382	1082
SbAGO1b	Sb09g000530	359137..365358	1109
SbAGO1c	Sb04g038420	67826355..67833897	1028
SbAGO1d	Sb10g031030	60700868..60707613	1016
SbAGO2	Sb06g028510	57283794..57289457	1092
SbAGO5a	Sb02g005150	5997121..6005181	1036
SbAGO4a	Sb03g011020	12166168..12173913	900
SbAGO4b	Sb09g030910	59524439..59531568	909
SbAGO5b	Sb01g004920	4014713..4023793	1067
SbAGO5c	Sb01g011870	10761650..10768577	1255
SbAGO5d	Sb01g011880	10774347..10780713	1087
SbAGO7	Sb01g032060	54885260..54889344	1033
SbAGO18	Sb02g032980	67564010..67569619	1044
SbAGO10	Sb10g023230	51757522..51763765	975

^(a)^Protein lengths were obtained from Phytozome annotations.

**Figure 6:**
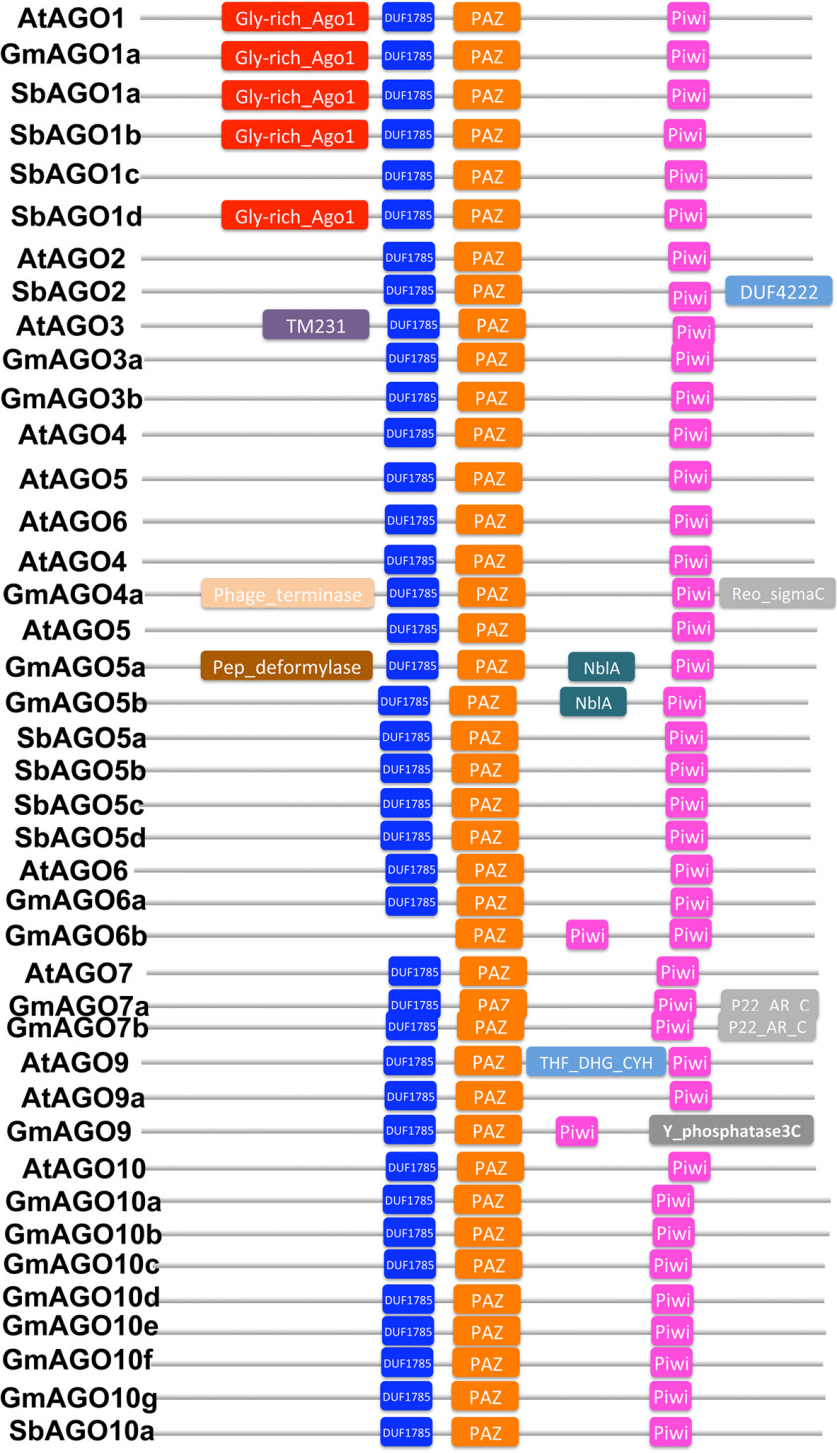
**Domain compositions of AGO genes in soybean, sorghum, and Arabidopsis.** All AGO genes have an N-terminal DUF1785 domain, a PAZ domain, and a C-terminal PIWI domain, except GmAGO6b, which misses the DUF1785 domain but has two PIWI domains. AGO1s have an additional Glycine-rich region on the N-terminus.

The domain combinations of these AGO proteins in Arabidopsis, soybean, and sorghum are shown in Figure 6. The PAZ and MID domains bind the 3′-end and 5′-phosphate of RNAs, respectively [47,48]. The PIWI domain has a similar structure as RNaseH and is responsible for the target mRNA cleavage. All the soybean and sorghum AGO proteins contains these four domains except for SbAGO6b, which does not have the N-terminal DUF1785 domain but possesses two tandem PIWI domains. The active site of one PIWI domain responsible for RNA cleavage often carries a conserved metal-chelating Asp–Asp–His (DDH) motif, which are correspond to D760, D845, and H986 of AtAGO1 [49]. Furthermore, a conserved histidine at position 798 of AGO1 in Arabidopsis has been shown to be essential for AGO cleavage activity [50]. Protein sequence alignment of all new discovered AGOs reveals that 10 soybean AGOs and 11 sorghum AGOs have the conserved DDH/H798 motifs (Table 6). In five soybean AGOs (GmAGO4a, GmAGO4b, GmAGO4c, GmAGO6a, and GmAGO9) and two sorghum AGOs (SbAGO4a and SbAGO4b), only the H798 is replaced by alanine, proline, or serine in the motif (Table 6). The histidine residue in the DDH motif is missed in GmAGO5b, GmAGO10g, and GmAGO10e, and replaced by aspartic acid in GmAGO3a, GmAGO3b and SbAGO2 (Table 6). GmAGO6b and AGO10g replace the second aspartic acid with alanine or lysine and AGO10g misses the third histidine in DDH motif (Table 6). Alterations in the catalytic motif in these AGOs indicate that they may not cleave targets. It has been shown that some of AGOs with the DDH motif do not have cleavage activity [51]. Thus, it needs to be verified whether AGOs with DDH motifs in sorghum and soybean have the cleavage activity.

**Table 6:** DDH/H motifs in AGO genes

**Soybean**	Motif*	**Sorghum**	Motif*
GmAGO1b	DDH/H	SbAGO1b	DDH/H
GmAGO1a	DDH/H	SbAGO1a	DDH/H
GmAGO3b	DDD/N	SbAGO1d	DDH/H
GmAGO3a	DDD/H	SbAGO1c	DDH/H
GmAGO4b	DDH/P	SbAGO5b	DDH/H
GmAGO4a	DDH/P	SbAGO5a	DDH/H
GmAGO4c	DDH/A	SbAGO5c	DDH/H
GmAGO5b	DD-/H	SbAGO5d	DDH/H
GmAGO5a	DDH/H	SbAGO4a	DDH/P
GmAGO6a	DDH/S	SbAGO4b	DDH/A
GmAGO6b	DAH/M	SbAGO18	DDH/H
GmAGO7a	DDH/H	SbAGO10a	DDH/H
GmAGO7b	DDH/H	SbAGO7	DDH/H
GmAGO9	DDH/S	SbAGO2	DDD/H
GmAGO10b	DDH/H		
GmAGO10a	DDH/H		
GmAGO10c	DDH/H		
GmAGO10g	DK-/-		
GmAGO10d	DDH/H		
GmAGO10e	DD-/H		
GmAGO10f	DDH/H		

*Motifs show the residues in soybean and sorghum AGO proteins that are correspond to D760, D845, H986/H798 of AtAGO1. The symbol “-” means the corresponding residue is missed.

### Experimental validation

**Figure 7:**
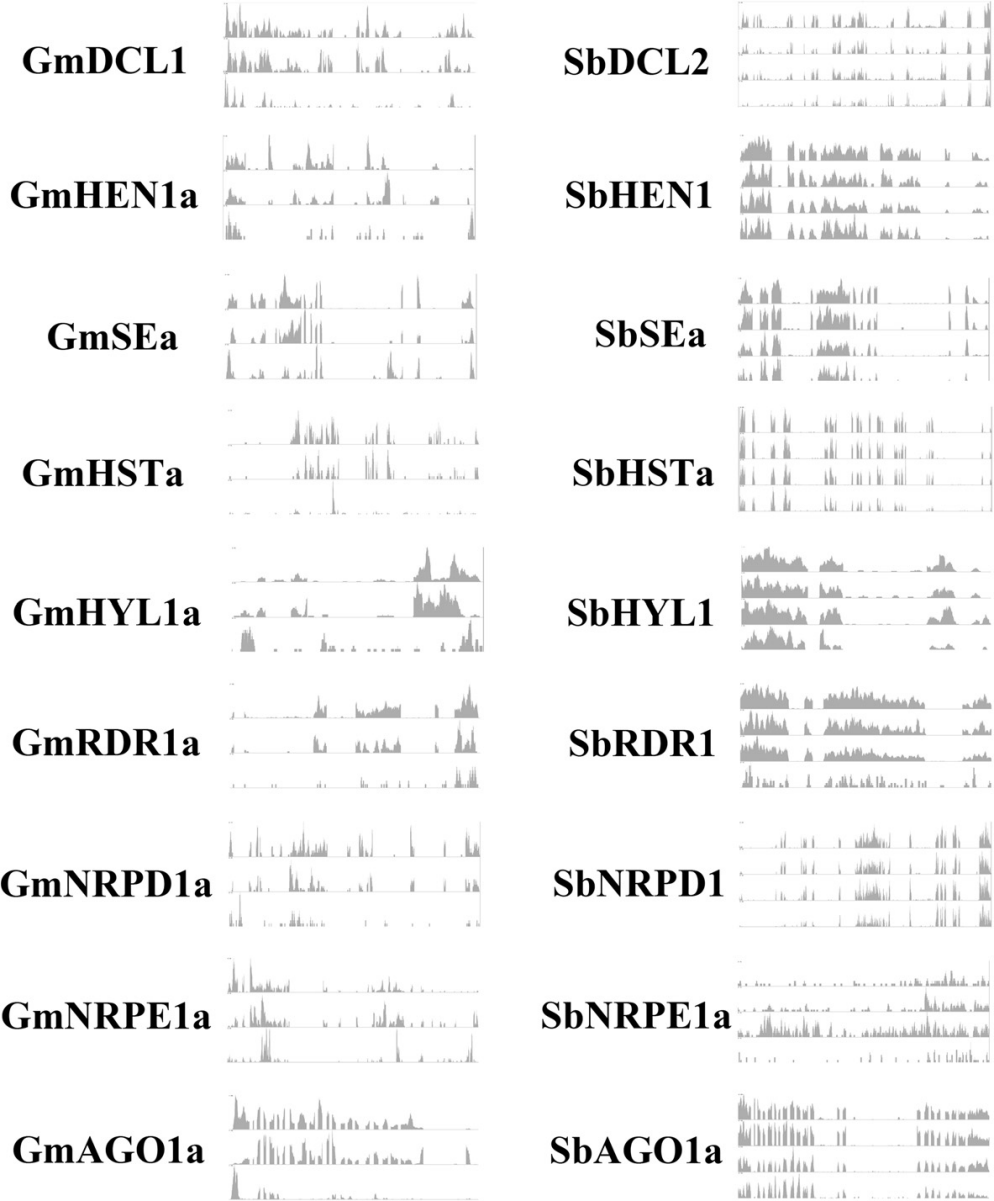
**The normalized depth of aligned RNA-seq reads in gene areas for eighteen discovered genes are shown.** The data from three libraries, SRX062333 (floral bud), SRX113962 (cotyledons), and SRX265552 (seeds), are displayed for soybean and four libraries, SRX080311 (root), SRX080321 (shoot), SRX080322 (shoot), SRX080323 (shoot), SRX099022 (early inflorescence), and SRX099184 (embryo) for sorghum. The y-axis for read depth is set for 200, but the gene lengths are various. The detailed RNA-seq data analysis results for all discovered genes are shown in Additional file 1: Table S1.

To confirm the expression of these RNA silencing components, we collected RNA-seq data from Sequence Read Archive (SRA), and analyzed these RNA-seq data to get the gene expression profiles for these new identified genes. According the numbers of mapped short reads, most identified genes have many mapped reads in different tissues and some of them even have very large numbers of mapped RNA-seq reads. Figure 7 shows the RNA-seq signals for some discovered genes, and detailed results of RNA-seq data analysis for all genes are shown in Additional file 1: Table S1. To further ascertain the RT-results, we searched those discovered RNA silencing components against the dbEST database [52] and PlantGDB [53] for expressed sequence tags (ESTs). We found the presence of ESTs of these genes in different tissues of soybean and sorghum. (Please see the Additional file 2: Table S2.) To further confirm these RNA silencing components in sorghum and soybean are indeed expressed, reverse transcription PCR (RT-PCR) was conducted. RT-PCR was performed on RNAs from inflorescence as templates using oligo dT primers. The resulting cDNA then was subjected to PCR using gene specific primers. RT-PCR identified the transcripts of these predicted RNA silencing components. Please see the Additional file 3: Figure S1 for RT-PCR results for those genes. The results of RT-PCR agree with RNA-seq data analysis results.

## Discussions

DCL is the essential component for miRNA and siRNA biogenesis [54]. Although animals encode one DCL for the generation of both miRNAs and siRNAs, plants evolve four DCL groups [54]. These DCLs have overlapping and diversified functions in miRNA and siRNA biogenesis [54]. Both sorghum and soybean possess four DCL families, which further supports the notion that expansion of DCL family members in monocots and dicots happens after divergence between animal and plants [54]. Sorghum has two DCL3 paralogs, DCL3a and DCL3b, which have low similarity to each other, whereas soybean encodes one DCL3. This result is consistent with the hypothesis that the DCL3 paralog in monocots was generated after divergence between monocots and dicots [55]. OsDCL3a acts in non-canonical long miRNA biogenesis and 24 ra-siRNA biogenesis, whereas OsDCL3b functions in phased 24-nt siRNA biogenesis, indicating that the function of DCL3 paralogs is diversified [56]. Because of the high similarities of SbDCL3a to OsDCL3a and SbDCL3b to OsDCL3b, SbDCL3a/b most likely have different functions in the small RNA pathway.

In Arabidopsis, DCL1, SE, TOUGH and HYL1 form a complex to process pri-miRNA in nucleus to generate miRNA duplex that are methylated by HEN1 and exported into cytoplasm by HST [4-6,12,13,15]. The identification of DCL1, HYL1, SE, HEN1, and HST homologs in sorghum and soybean suggests that the biogenesis processes of miRNAs in them are similar to that of Arabidopsis. It is noted that in sorghum, the paralogs of HYL1, SE, HEN1, and HST are less similar to each other, but each has a closely related homolog in rice. This indicates that the duplication may occur before divergence between rice and sorghum about 50–70 million years ago [55]. However, one can note that SEs in both soybean and sorghum have three paralogs each, which is more than other components in soybean/sorghum and SE in Arabidopsis do. This indicates the selective duplication for SEs in soybean and sorghum, besides whole genome duplication.

RDR is essential for siRNA biogenesis as well [8]. Studies from Arabidopsis, rice, and maize have shown that plants possesses four groups of RDRs: RDR1, RDR2, RDR3 and RDR6. RDR2 from Arabidopsis and maize (MOP2), RDR6 from Arabidopsis and rice are required for ra-siRNA and ta-siRNA biogenesis, respectively [45,46]. Recently, it was shown that RDR6 acts redundantly with RDR1 in viral-derived siRNA biogenesis [57]. The function of RDR3 family is currently unknown yet. Corresponding RDR1, RDR2, RDR3, and RDR6 homologs for both soybean and sorghum are identified, which further supports the notion that the RDR gene family in plants is derived from a common ancestor.

The putative largest subunit and the second largest subunit of Pol IV and PolV, which are required for ra-siRNA-mediated DNA methylation, are discovered from soybean and sorghum. This agrees with the notion that Pol V and Pol IV are plant specific polymerases. In maize, lack of Pol IV and Pol V causes development defects [58], whereas in Arabidopsis, the *nrpd* and *nrpe* mutants appear to grow normally. It is interesting to further test whether Pol IV and Pol V are necessary for the development of soybean and sorghum.

AGO is the effector protein for small RNA-mediated silencing [1]. It is proposed that both plants and animals encode multiple AGOs to meet the diversified functions of small RNA silencing [1]. Like rice, maize, and Arabidopsis, both soybean and sorghum encode three subfamilies of AGO proteins, indicating that small RNA functions are conserved in higher plants. Soybean encodes seven AGO10 paralogs. Among of them, GmAGO10a/b/c share high similarity to each other, while GmAGO10d/e/f/g are clustered. The similarity of these two groups of GmAGO10 is relatively low, which indicates that their functions might be different. They might regulate the functions of different miRNAs. In Arabidopsis, AGO10 has been shown to regulate the function of miR166/165 [28,29].

The identification of these putative RNA silencing components would give insight on small RNA pathways in soybean and sorghum. However, the exact function and contribution of individual component of RNA silencing machinery needs to be further examined because their functions may be diverse among different plant species.

## Conclusions

Small RNA-mediated gene silencing is an important mechanism to regulate gene expression and genome stability in plants. The available sorghum and soybean genome information enable the identification of components that may involve in small-RNA mediated gene silencing in soybean and sorghum [59,60]. The gene families, including DCL, HEN1, SE, HYL1, HST, RDR, NRPD1, NRPD2/NRPE2, NRPE1, and AGO, in soybean and sorghum were identified. RNA-seq, EST and RT-PCR analysis confirmed the expression of these candidate genes. In soybean, the similarities among paralogs are very high, which is consistent with the hypothesis that there have been 1–2 rounds of genome duplication in soybean since the separation of homolog sequences between soybean and Arabidopsis approximately 90 million years ago [55]. Based on the knowledge of their counterparts in Arabidopsis, putative functions to these genes are annotated.

## Methods

### Genome sequence data

We collected soybean (Gmax 189) and sorghum (v1.4) genome sequences from Phytozome (v9.0) (http://www.phytozome.net/), and Arabidopsis sequences from TAIR (10) (http://www.arabidopsis.org/). The total numbers of genes are 55787, 35386, and 29448 for soybeans, sorghum, and Arabidopsis, respectively.

### Identification of miRNA components

HMM analysis was used to search for DCL, AGO, and RDR genes encoded in the soybean and sorghum genomes, besides searching homolog in Arabidopsis with TBLASTN. DCL proteins have domains of DExD-helicase, helicase-C, Duf283, PAZ, RNase III, and double-stranded RNA-binding (dsRB). AGOs have PAZ, MID, and PIWI domains. RDRs have a conserved RDRP domain. The HMM profiles of domains in DCL, AGO and RDR families are obtained from the Pfam database. With the HMM profiles, the corresponding conserved sequences of DCL, AGO, and RDR proteins are extracted by HMMER [61]. These conserved sequences are adapted to search for all predicted DCL, AGO and RDR genes. Protein sequences of all candidate genes were also aligned against Arabidopsis genome with BLASTP program (cutoff E-value = 0.001). The other genes, HEN1, SE, HYL1, and HST, which have only one gene in Arabidopsis, were screened against soybean and sorghum genomes with TBLASTN program (cutoff E-value = 0.001) to find the candidate genes.

### Phylogenetic analysis

Clustal-W was used for multiple sequence alignments. Phylogenetic analysis was performed with the PhyML and MEGA v5.0 programs by the maximum-likelihood method with 500 bootstrap replicates.

### RNA-seq data analysis

RNA-seq data for soybean and sorghum were obtained from SRA (http://www.ncbi.nlm.nih.gov/Traces/sra/), and the accession numbers of these RNA-seq data are SRX062333 (floral bud), SRX113962 (cotyledons), and SRX265552 (seeds) for soybean and SRX080311 (root), SRX080321 (shoot), SRX080322 (shoot), SRX080323 (shoot), SRX099022 (early inflorescence), and SRX099184 (embryo) for sorghum. After preprocessing the RNA-seq data, the short reads were mapped against the *G. max* 189 genome and *S. bicolor* v1.4 genome sequences using Tophat (v1.3.2) [62], allowing up to two mismatches. The numbers of reads in genes were counted by HTSeq-count tool (Anders, 2010) [63] with the “union” resolution mode, and they are normalized with scaling the total count of mapped reads to 10 million reads. For each gene, the numbers of mapped reads per kilobase of exon per million mapped reads (RPKM) is shown as well.

### EST expression analysis

To estimate the expression profiles, all miRNA components are searched against the dbEST database [52] (http://www.ncbi.nlm.nih.gov/dbEST) and PlantGDB [53] (http://www.plantgdb.org) with MEGABLAST (cutoff E-value = 10^-10^).

### RT-PCR analysis

Total RNAs from inflorescences of soybean or sorghum was extracted as described in the work of Yu *et al*. [64]. After treatment with DNase I, 5 μg RNA was reverse transcribed (RT) by the Superscript III reverse transcriptase (Invitrogen) using an oligo-T18 primer to generate cDNAs at 50°C for 1 hour. The resulting cDNAs was used as templates to perform PCR amplification with primers listed in Additional file 4: Table S3. PCR was performed for 32 cycles (94°C for 30 seconds, 55°C for 30 seconds, and 72°C for 60 seconds). Total RNAs were extracted from inflorescences of soybean or sorghum. Reverse transcription was performed using an Oligo-T primer. The amplification of *UBIQUITIN 5* (*UBQ5*) was used as a loading control.

## Competing interests

The authors declare that they have no competing interests.

## Authors’ contribution

XL, BY, and CZ desigend the experiment. XL conducted the experiment. TL, YD, and CZ conducted the bioinformtic study and data analysis. YD conducted the RNA-seq data analtysis. CZ and BY supervised the whole project and drafted the manuscript. All authors read and approved the final manuscript.

## Supplementary Material

Additional file 1: Table S1 Raw and normalized numbers of mapped RNA-seq reads for all discovered genes in different tissues of soybean and sorghum. RNA-seq data were obtained from SRA and TopHat was used to map short reads to soybean and sorghum genome sequences. The number of reads aligned in one gene reflects the gene expression level in the given tissue. Click here for file

Additional file 2: Table S2 Numbers of ESTs for all discovered genes in soybean and sorghum. ESTs from dbEST and PlantGDB, including PlantGDB-assembled unique transcripts (PUTs), were mapped to all identified genes with MEGABLAST. The numbers of aligned ESTs are related to the gene expression, and Symbols of “X” for different tissues mean that some ESTs are identified in corresponding tissues. Click here for file

Additional file 3: Figure S1 Detection of predicted genes involved in the RNA silencing pathway in soybean (A) and sorghum (B). The transcripts are detected by RT-PCR. Amplification of *UBIQUITIN5* (*UBQ5*) with or without RT (-RT) is shown as a control. Click here for file

Additional file 4: Table S3 List of primers that were used for PCR amplification. Click here for file

## Declarations

The work is supported by funding under CZ’s startup funds from University of Nebraska, Lincoln, NE, USA and BY’s grant from Nebraska Soybean Board, NE, USA.
