# Supplementary figures and images for: Identification of RNA silencing components in soybean and sorghum
Source: BMC Bioinformatics. 2014 Jan 4;15:4. doi: 10.1186/1471-2105-15-4 (PMC3882329; doi:10.1186/1471-2105-15-4)

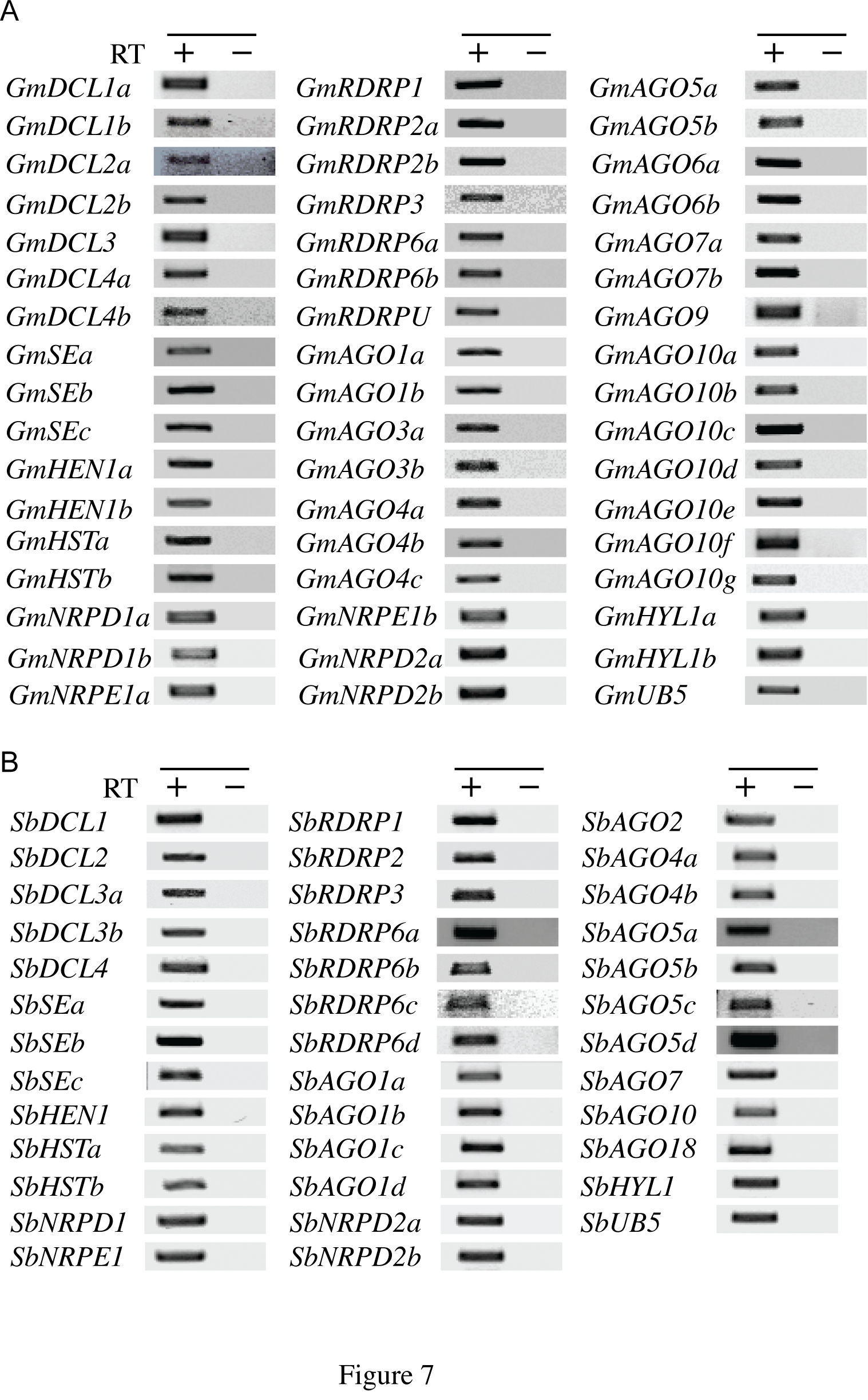

Supplement: Additional file 3: Figure S1 — Detection of predicted genes involved in the RNA silencing pathway in soybean (A) and sorghum (B). The transcripts are detected by RT-PCR. Amplification of UBIQUITIN5 (UBQ5) with or without RT (-RT) is shown as a control. [file 1471-2105-15-4-S3.tiff]
