# Supplementary material for: Identification of RNA silencing components in soybean and sorghum
Source: BMC Bioinformatics. 2014 Jan 4;15:4. doi: 10.1186/1471-2105-15-4 (PMC3882329; doi:10.1186/1471-2105-15-4)
Supplement: Additional file 4: Table S3 — List of primers that were used for PCR amplification. [file 1471-2105-15-4-S4.docx]

**Supplemental Table 3.** Primers used in this study

| Name | Sequence(5’–3’) |
| --- | --- |
| **DCL primers** |  |
| GmDCL1a LP | GCAGCTTTGCAAAATGATGA |
| GmDCL1a RP | TGATTAGTGCCTGCACCTTG |
| GmDCL1b LP | GCAGCTTTGCAAAATGATGA |
| GmDCL1b RP | TGATTAGTGCCTGCACCTTG |
| GmDCL2a LP | CTGCGAAAGCCTTCTCCTTA |
| GmDCL2a RP | GATGCAGGCATAAGGGTGTT |
| GmDCL2b LP | CTTGGTGTTTGGTTGGCTTT |
| GmDCL2b RP | GGCTGTAATGACCCTTTCCA |
| GmDCL3 LP | CTGTCTGTGGAAGGGGGTTA |
| GmDCL3 RP | CTCCCCATGCACCACTTAAT |
| GmDCL4a LP | TTGGTGGACAGTGGCAATAA |
| GmDCL4a RP | TGCATTGGAAGGCAATGTTA |
| GmDCL4b LP | AGTTGGCTATCAAGCGCAAT |
| GmDCL4b RP | TTGAATGCCAATCCATGAAA |
| SbDCL1 LP | AGGGTTATCTGGGTCCCTTG |
| SbDCL1 RP | TGCTCTCCACCCTTCTCATT |
| SbDCL2 LP | TACACTGTTGCCAGCGAATC |
| SbDCL2 RP | CGCAGCTTTCCTTGTTTGAT |
| SbDCL3a LP | TGGGAAAACCATGTGACAGA |
| SbDCL3a RP | TGGATGTGATACGGCTTCAA |
| SbDCL3b LP | AATTGACAAACGCCTCCAAC |
| SbDCL3b RP | TTTTAGGGGTGAGTGCATCC |
| SbDCL4 LP | AAGCTTCTCGGTGGAGCATA |
| SbDCL4 RP | GCTTTCAAAAGTGGCTGCTC |
| **RDRP primers** |  |
| GmRDRP1a LP | GAAGCCTGACAAACCCACAT |
| GmRDRP1a RP | GCATCCCTCCTTTTGTTGAA |
| GmRDRP1b LP | TAGAAGAGTGGCGAGGCAAT |
| GmRDRP1b RP | GGCATCACGACCTTGAGAAT |
| GmRDRP2a LP | GCCTTCCTTGAGACAGCATC |
| GmRDRP2a RP | ATAATGGCTGCGGTGATAGG |
| GmRDRP2b LP | GCCTTCCTTGAGACAGCATC |
| GmRDRP2b RP | GGCAATAATAGCTGGGGTGA |
| GmRDRP3 LP | ATTTGCTGAGGATGGGAGTG |
| GmRDRP3 RP | AATTGGGCAACAACATGTGA |
| GmRDRP6a LP | TTATGGATGAAGGCATGCAA |
| GmRDRP6a RP | CCCATCCTAGCAGCACATTT |
| GmRDRP6b LP | TGATTTCATGGGGAAAGAGC |
| GmRDRP6b RP | TTTTGGCATGGACCAAATCT |
| SbRDRP1 LP | GACAGTGCCGAGTCTGTCAA |
| SbRDRP1 RP | CCTTAACCAAGCATCCCAAA |
| SbRDRP2 LP | GCAGCTGACTTATGCACCAA |
| SbRDRP2 RP | GGCAGTCAACAAGAGGAACC |
| SbRDRP3 LP | TCCAGACCGTGTTGAAATGA |
| SbRDRP3 RP | GCTAACATCAGAGGGCTTGG |
| SbRDRP6a LP | TAGAATGCCACCATCCCTTC |
| SbRDRP6a RP | CTGTCACAAGCTCTGCTTCG |
| SbRDRP6b LP | TGTTCCTCGTGAATGCTCTG |
| SbRDRP6b RP | TAGAGCGCTCCACTTCTGGT |
| SbRDRP6c LP | CCTTCCACCTGATCGACACT |
| SbRDRP6c RP | CGGAAACCACAGGTCTTCAT |
| SbRDRP6d LP | TTGCAGCTATTGCTGTGGAC |
| SbRDRP6d RP | GAAAACTCAAGGCACCAGGA |
| **AGO primers** |  |
| GmAGO1a LP | AGAGGAGTGAACCGTGCTGT |
| GmAGO1a RP | AAGGACCTGCAAAGCCTCTT |
| GmAGO1b LP | CCCAAGTTCATAGGCAGGAA |
| GmAGO1b RP | AGCAAAGAGCCTTGTGTGGT |
| GmAGO3a LP | TCACTGGAATCTTGCTGGAA |
| GmAGO3a RP | AGAAATTGCGGGTGAACTTG |
| GmAGO3b LP | AGTGGCAACCAAGGTCAAAC |
| GmAGO3b RP | TGGTTCACACGGAGATAGCA |
| GmAGO4a LP | TGCTTCAAAAGGGACAAAGC |
| GmAGO4a RP | GCAAAATCCTTCCCATTCAA |
| GmAGO4b LP | CTCCACGGTTGAAATTTGGT |
| GmAGO4b RP | AGCCCCAGGAAGTTTTGACT |
| GmAGO4c LP | AATGATGGGCATTTCTTCCA |
| GmAGO4c RP | ACGTCGCATCCTCTTTCTGT |
| GmAGO5a LP | TTTTGTGATCGTGCTGAAGG |
| GmAGO5a RP | GAAAAACGATCTTCCCACCA |
| GmAGO5b LP | GCTGGTGTTGTGGAACATTG |
| GmAGO5b RP | TGGAGTCTTCCTTGGTTTGC |
| GmAGO6a LP | CTGCTGAACCTCCAACATCA |
| GmAGO6a RP | CAAACCTTTTGCCACCAAGT |
| GmAGO6b LP | CTGTTGAAAGCAAGGGCATT |
| GmAGO6b RP | CCTCATGAAGGCTTCCGTTA |
| GmAGO7a LP | TCTCAGGTGCTACTCCAGCA |
| GmAGO7a RP | AATCATCACCCTCGTTGCTC |
| GmAGO7b LP | CGAAAAACCATTGTCGAAGG |
| GmAGO7b RP | ATGAATCTGGGGACATTGGA |
| GmAGO9 LP | TGTGGTGGACTTCCTTCTCC |
| GmAGO9 RP | TTTTGGTTTGCCAACATTGA |
| GmAGO10a LP | GGCCCCTTGGCTTAAATATC |
| GmAGO10a RP | TTGCTCCCTGACACATGGTA |
| GmAGO10b LP | TTCCTGCTCCTTGGCTTAAA |
| GmAGO10b RP | CTCCCTGCCACATGGTAAAC |
| GmAGO10c LP | AACCACAGGGACAGGAACAG |
| GmAGO10c RP | ATATAGAACCGCGCTCGAAA |
| GmAGO10d LP | ACCTTTACACTGCCGGTTTG |
| GmAGO10d RP | ACCCAACTGCTGTGGTTTTC |
| GmAGO10e LP | ATGCAAAGGAGTTTGGCATC |
| GmAGO10e RP | TGACTGAATTCCATGCCTGA |
| GmAGO10f LP | ACACTGACTTGGGGATGAGG |
| GmAGO10f RP | AGTCGCAAGCTCCCTCAATA |
| GmAGO10g LP | TCCCTATGCCAAGGAGTTTG |
| GmAGO10g RP | ATTCCATGCCTGAGATTTGG |
| SbAGO1a LP | GCCTCACTTCACAAGCAACA |
| SbAGO1a RP | TGTCTTTCTCACGCTCATGG |
| SbAGO1b LP | ATATCAGGGACGAGGTGGTG |
| SbAGO1b RP | TTCTTGGCTCGTGGAAGACT |
| SbAGO1c LP | GGCCAAGGTCAAGCTTACAG |
| SbAGO1c RP | TGGGCGATGAGGAAACTTAC |
| SbAGO1d LP | CATCATCAAGGAGCTGGTCA |
| SbAGO1d RP | AGTTCCCGCAGCACAATATC |
| SbAGO2 LP | ACTGAACAGGAGGCAACTGG |
| SbAGO2 RP | CCTTGCTCTTGCTCAAATCC |
| SbAGO4a LP | AGAAATGGGCAGTGGTCAAC |
| SbAGO4a RP | CTCCAGGAAGCTTGGTTTTG |
| SbAGO4b LP | AGACATATCGCGCTGAGCTT |
| SbAGO4b RP | TCTCCGTTTCTTCGCCTCTA |
| SbAGO5a LP | GGTGGAGAAGAAGCTGTTCG |
| SbAGO5a RP | TGGTTTTTCCGATTCCATGT |
| SbAGO5b LP | GTCATCCCAGCGAGTTTGAT |
| SbAGO5b RP | ATCGGTGCTCTCTTGTTCGT |
| SbAGO5c LP | CTGCAGACCTCGAAGGATTC |
| SbAGO5c RP | CTCGGCCACTACAACCTTTC |
| SbAGO5d LP | CAATACTTGGATGGGCGACT |
| SbAGO5d RP | CCAGCCAAGAACTGCCTAAG |
| SbAGO7 LP | GAGGAGGGAGGTGGAAAAAG |
| SbAGO7 RP | AAGCTCCATTGGCACATAGC |
| SbAGO10 LP | GAAGTACCGTGGCACTGGAT |
| SbAGO10 RP | TCTTAGCCTGTTCCCAGCAT |
| SbAGO18 LP | CGTCGTCAAGGCTAACCACT |
| SbAGO18 RP | CTTGATGGCGACCCTGTACT |
| **Other primers** |  |
| GmHEN1a LP | TCCTGAGGTGGAGTCAAACC |
| GmHEN1a RP | GGAGGCATCTTCAAAACCAA |
| GmHEN1b LP | TGGGGATGTGGCATTAAGTT |
| GmHEN1b RP | ATAGCCGGGTTCAACATCAG |
| GmSEa LP | TGAAAGGCAAATGGGAAATC |
| GmSEa RP | GCATCTGTCGAGGATCAGGT |
| GmSEb LP | GAGCTAAGGGCTGCACAAAG |
| GmSEb RP | GGGAACCATCAGGCCTATCT |
| GmSEc LP | AGGGCTTTAGGCATGTGAGA |
| GmSEc RP | GCTCTGGGTGTTTGAGCTTC |
| GmHSTa LP | AGGATACGCTGAACCCAATG |
| GmHSTa RP | CTTGCTGAGCATGGACAAAA |
| GmHSTb LP | GTCTGGCCCTTCCAACACTA |
| GmHSTb RP | TGGGGAGAGACATCAAAACC |
| GmHYL1a LP | TCTGGCCTCCTTGAAAAGAA |
| GmHYL1a RP | TCCACACTTGGTTCCGGTAT |
| GmHYL1b LP | GGCCGAGCATCAGTATTTTC |
| GmHYL1b RP | TCTTAGGCTTTGGGGGATTT |
| GmNRPD1a LP | GTGACCATGCAGACCTTCCT |
| GmNRPD1a RP | AACTGGTTGACCCAAAGCAC |
| GmNRPD1b LP | CTTCTGAGGCTTCTGGTTGG |
| GmNRPD1b RP | TGAAATTACAAGCCCGCTCT |
| GmNRPE1a LP | GGGTTCAAATGCTGGTGACT |
| GmNRPE1a RP | CAGTGCCCATTTTGGTTTCT |
| GmNRPE1b LP | TTCACTCAGGGAGGGCTCTA |
| GmNRPE1b RP | CAGCAAGTGACTGCGGATAA |
| GmNRPD2a/NRPE2a LP | TTGATGATGATGGCTTTCCA |
| GmNRPDa/NRPE2a RP | CCAAAACTCCCTTTTGTCCA |
| GmNRPD2b/NRPE2b LP | TTGATGATGATGGCTTTCCA |
| GmNrpd2b/NRPE2b RP | CCAAAACTCCCTTTTGACCA |
| SbHEN1 LP | GTGCTTGCTTGTCATGGAGA |
| SbHEN1 RP | AGCAAAGGAGATCTCGTGGA |
| SbSEa LP | ACTTACCGCCAAGGACAAAA |
| SbSEa RP | CTCCGTCTTCCTCTGTCTGG |
| SbSEb LP | GGGATGAGCGTGGTAATCAT |
| SbSEb RP | CAACACCTCTCCCTGCTTTC |
| SbSEc LP | AAAATGCGGGATGAGAATTG |
| SbSEc RP | ACACATGGGGAGAAGTGGAG |
| SbHSTa LP | CCCTTTTGTGGTGCTTTGAT |
| SbHSTa RP | TTCGCATATGTTGCTTTTGC |
| SbHSTb LP | TGCATCTCCAGAGTTGATGG |
| SbHSTb RP | CTGCGAAGGAAATTCCACAT |
| SbHYL1 LP | ATAGTGCAACCGGAGTCACC |
| SbHYL1 RP | GCTTCTTGCCTTGTGGTTTC |
| SbNRPD1 LP | CCTGCACCTTCTAACCAAGG |
| SbNRPD1 RP | GCCATCAAAATCTCCCAGAA |
| SbNRPE1a LP | GATTCCCTGCTGCATGATTT |
| SbNRPE1a RP | ATTCTGCATTGGCAAAGTCC |
| SbNRPD2a/NRPE2a LP | AAAGCGAGTGCTTGTTCGAT |
| SbNRPD2a/NRPE2a RP | AACTGGCATTGTCGCATACA |
| SbNRPD2b/NRPE2b LP | CCACAGCATCTGTTGACGTT |
| SbNRPD2b/NRPE2b RP | CCAAACTTAACTCCGCCAAA |
